# Personalizing Dual-Target Cortical Stimulation with Bayesian Parameter Optimization Successfully Treats Central Post-Stroke Pain: A Case Report

**DOI:** 10.3390/brainsci12010025

**Published:** 2021-12-26

**Authors:** Evan M. Dastin-van Rijn, Seth D. König, Danielle Carlson, Vasudha Goel, Andrew Grande, Donald R. Nixdorf, Sarah Benish, Alik S. Widge, Ziad Nahas, Michael C. Park, Tay I. Netoff, Alexander B. Herman, David P. Darrow

**Affiliations:** 1 Department of Biomedical Engineering, University of Minnesota, Minneapolis, MN 55455, USA; dasti006@umn.edu (E.M.D.-v.R.); tnetoff@umn.edu (T.I.N.); 2 Department of Psychiatry and Behavioral Sciences, University of Minnesota Medical Center, Minneapolis, MN 55454, USA; koeni117@umn.edu (S.D.K.); carl1492@umn.edu (D.C.); awidge@umn.edu (A.S.W.); znahas@umn.edu (Z.N.); herma686@umn.edu (A.B.H.); 3 Department of Neurosurgery, University of Minnesota Medical Center, Minneapolis, MN 55455, USA; grande@umn.edu (A.G.); mcpark@umn.edu (M.C.P.); 4 Department of Anesthesiology, University of Minnesota Medical Center, Minneapolis, MN 55455, USA; vgoel@umn.edu; 5 Department of Diagnostic & Biological Sciences, School of Dentistry, Minneapolis, MN 55455, USA; nixdorf@umn.edu; 6 Department of Radiology, Medical School, Minneapolis, MN 55455, USA; 7 Department of Neurology, University of Minnesota Medical Center, Minneapolis, MN 55455, USA; smbenish@umn.edu

**Keywords:** cortical stimulation, stroke, chronic pain, electrophysiology, optimization

## Abstract

Central pain disorders, such as central post-stroke pain, remain clinically challenging to treat, despite many decades of pharmacological advances and the evolution of neuromodulation. For treatment refractory cases, previous studies have highlighted some benefits of cortical stimulation. Recent advances in new targets for pain and the optimization of neuromodulation encouraged our group to develop a dual cortical target approach paired with Bayesian optimization to provide a personalized treatment. Here, we present a case report of a woman who developed left-sided facial pain after multiple thalamic strokes. All previous pharmacologic and interventional treatments failed to mitigate the pain, leaving her incapacitated due to pain and medication side effects. She subsequently underwent a single burr hole for placement of motor cortex (M1) and dorsolateral prefrontal cortex (dlPFC) paddles for stimulation with externalization. By using Bayesian optimization to find optimal stimulation parameters and stimulation sites, we were able to reduce pain from an 8.5/10 to a 0/10 during a 5-day inpatient stay, with pain staying at or below a 2/10 one-month post-procedure. We found optimal treatment to be simultaneous stimulation of M1 and dlPFC without any evidence of seizure induction. In addition, we found no worsening in cognitive performance during a working memory task with dlPFC stimulation. This personalized approach using Bayesian optimization may provide a new foundation for treating central pain and other functional disorders through systematic evaluation of stimulation parameters.

## 1. Introduction/Background

Central post-stroke pain (CPSP), also known as Dejerine-Roussy syndrome or thalamic pain syndrome, is a neuropathic pain syndrome that develops after a thalamic stroke. It is characterized by a period of numbness, followed by lasting and debilitating pain in the distribution of the body affected by the stroke. The pain typically fluctuates in intensity yet is constantly present and accompanied by altered temperature and touch sensation [1]. The pathophysiology underlying CPSP remains poorly understood, with a variety of putative mechanistic explanations proposed including changes in thalamic signaling deafferentation, hyperexcitation in damaged pathways, and imbalances in thalamo-cortical oscillatory “dialogue” [2].

CPSP remains one of the most intractable pain disorders. A variety of oral and intravenous drugs have been prescribed for treatment, including tricyclic antidepressants, anticonvulsants, and opioids, with limited response and frequent side effects [2]. Invasive neurostimulation therapies have also been attempted, including deep brain stimulation of the thalamus and periventricular gray matter and direct motor cortex stimulation. Neurostimulation efficacy rates range from 25% to 67% [2]. Unfortunately, none of these interventions has consistently proven to be long lasting, and efforts to optimize neurostimulation have not been systematic [3].

Since many of these reports were published, advancements in neuromodulation and its underlying technology have fueled innovation in novel targets, personalization, and closing the loop between electrophysiology and neurostimulation [4,5,6]. Recently, we have developed and tested a novel Bayesian optimization platform for objectively determining neurostimulation parameters based on patient feedback with forced binary choice [7]. This approach facilitates a methodology for continuous optimization that we hypothesized could aid with finding optimal parameters during a trial externalization for CPSP.

Multiple novel stimulation targets for central pain disorders have been proposed other than motor cortex stimulation. One area with particular promise is the dorsolateral prefrontal cortex (dlPFC) [8], with noninvasive TMS showing significant reductions in symptoms for patients with chronic neuropathic pain [9,10]. However, targeting the dlPFC with intracranial stimulation has not yet been tested for pain but has been successful in other indications, such as depression [11,12]. Because a second electrode can be placed through the same burrhole used for motor cortex stimulation, pairing two electrodes, one over dlPFC and the other over the M1 anatomical area, provides both convenient target selection and improved flexibility of cortical stimulation therapy for CPSP. 

This case report is the first description of the use of Bayesian parameter space optimization combined with electrophysiology to treat CPSP using dual cortical stimulation in motor cortex (M1) and dlPFC. We found that combined stimulation was the most effective approach, providing more relief than either single simulation site. Simultaneous electrophysiological recordings and quantitative behavioral assessment showed no indication of epileptiform activity or cognitive impairment. We believe this approach provides a new foundation for treating central pain disorders through systematic, personalized evaluation.

## 2. Case Presentation

### 2.1. Case History

**Figure 1:**
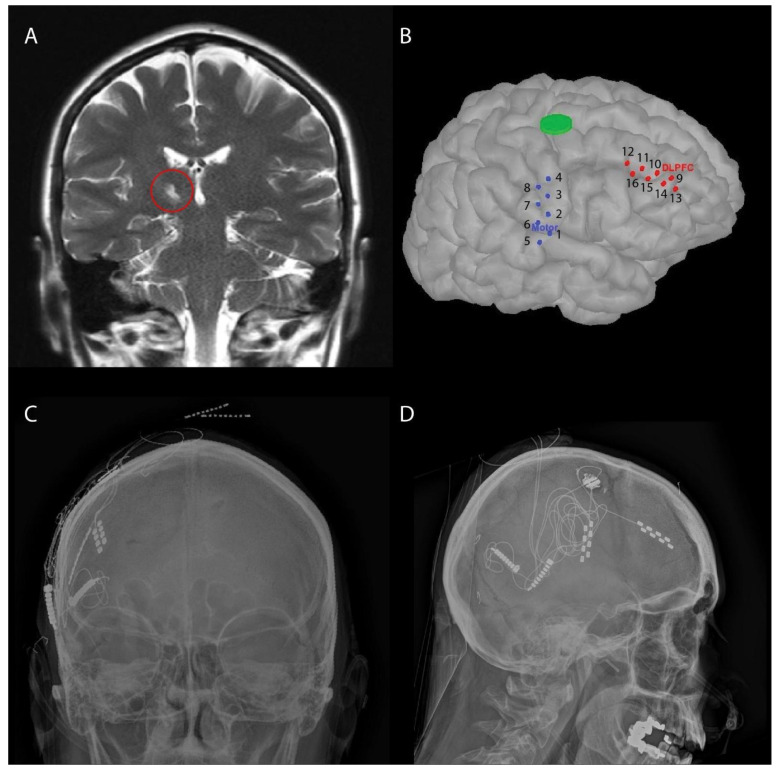
(**A**) T2 MR Coronal section of thalamic infarct (circled in red). (**B**) M1 and dlPFC electrode visualization by co-registration of the patient’s MRI and CT using SPM12 toolbox in Brainstorm [13,14], green circle is the burr hole location. (**C**) Anteroposterior X-ray projection of electrodes. (**D**) Lateral X-ray projection of the two Abbott Lamitrode 44 electrodes implanted on the patient’s right side. with the tunneled extensions seen superior and posteriorly.

A 45-year-old woman with a medical history of multiple right sided thalamic strokes due to paradoxical emboli and an 18-month history of CPSP presented to our multidisciplinary facial pain clinic. At the time of the strokes, she experienced significant deficits, including left-sided hemiparesis and hemianesthesia. Four years later, she developed left-sided facial pain. Her facial pain began as a sensation of cold that progressed into her jaw and then up to her forehead. She described it as much worse than any headache or migraine and compared it to constant electrical shocks, rating her pain intensity as being between an 8 to 10 on a 0–10 numerical rating scale, where 0 is no pain and 10 is as worst as imaginable. She tried multiple medications, including indomethacin (Indocin), cyclobenzaprine (Flexeril), lamotrigine (Lamictal), gabapentin (Neurontin), oxcarbazepine (Trileptal), onabotulinum toxin A (Botox), pregabalin (Lyrica), propranolol (Inderal), buspirone (Buspar), quetiapine (Seroquel), hydroxyzine, medical cannabis, erenumab (Aimovig), ubrogepant (Ubrelvy), several different opioid analgesics, and baclofen, as well as topical and subcutaneous local anesthetic. None resulted in substantial nor sustained pain relief. Her facial pain was debilitating and dominated every aspect of her life. An MRI of her brain demonstrated a significant focal infarct in the right thalamus (Figure 1A), and, given her history, we diagnosed her with CPSP. We offered her cortical stimulation focused on the right face region of M1 and dlPFC through a single burr hole, with inpatient externalization for trialing as a last resort therapy, with extensive discussions of the alternatives and risks. 

### 2.2. Surgical Procedure: Placement of Subdural Electrodes and Externalization

Prior to surgery, an MRI was obtained for identification of the face M1 and dlPFC targets (Figure 1A). Medtronic Stealth Neuronavigation (Medtronic, Dublin, Ireland) was used to plan the site of a burrhole superior to the M1 target along a trajectory to maximize coverage of the face portion of the motor cortex (Figure 1B). A 5-mm cutting burr was used to make this single burr hole, and the dura was sharply opened. Hemostasis was obtained, and two Lamitrode 44 electrodes (Abbott, Abbot Park, IL, USA) were placed at the target sites, which were confirmed using projections on lateral X-ray (Figure 1D). Intraoperative stimulation on the M1 electrode demonstrated evoked responses in orbicularis oculi under general anesthesia. A Guardian cranial burr hole cover (Abbott, Abbott Park, IL, USA) was used to secure the leads. Lead extensions were tunneled from a postauricular pocket to preserve the electrodes for later use, as shown in Figure 1C,D.

### 2.3. Externalization Trialing

On postoperative day 1, the broadest bipolar electrode pairs (electrodes 5–8 in M1 and 13–16 in dlPFC) were tested for a range of frequencies (10–50 Hz) and pulse widths (300 and 500 μs). We focused on determining safe amplitude ranges and baseline response to stimulation. Motor threshold was determined to be between 3.5–4.0 mA for 50 Hz, 300 μs stimulation. She described feelings of relief and “throbbing” aligned to changes in stimulation parameters. Simultaneous electrophysiological recordings on the electrodes indicated no abnormal neural or seizure-like activity during or after stimulation.

**Figure 2:**
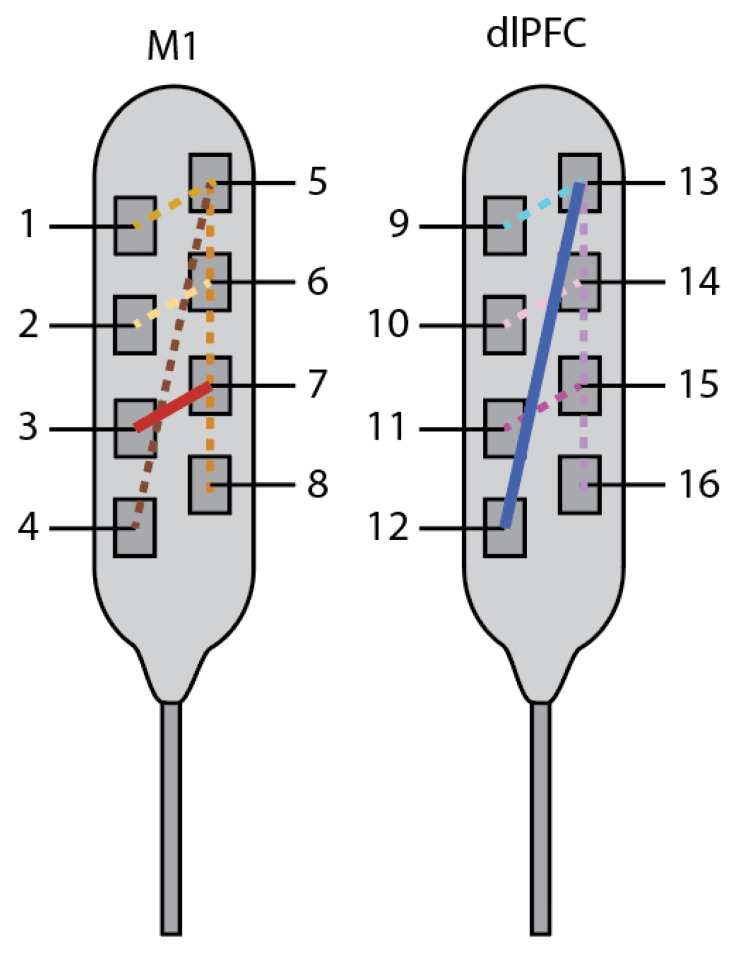
Day 1 and 2: parameter sweep over bipolar pairs (lines) for choice of stimulating contacts. Preferred stimulation electrodes were 3 and 7 for M1 (red solid line), and 12 and 13 for dlPFC (dark-blue solid line).

On the second day, we systematically evaluated spatial stimulation by including four additional bipolar pairs for each paddle with varying amplitude and frequency and a constant pulse width. (Figure 2). Preference and sensation of relief varied highly with the choice of parameters, with side effects, including twitching, numbness, difficulty speaking, perceptions of itchiness, pulsing, heat, cold, and a void of feeling. During this testing, her pain varied from 5–8/10. Based on her descriptions and self-reported feeling of relief, one bipolar contact pair was chosen on each paddle (3–7 in M1 and 12–13 in dlPFC). Tonic stimulation on M1 at 2.6 mA, frequency of 50 Hz, and pulse width of 150 μs was programmed for overnight testing. She rated her pain as a 4/10 that evening. 

**Figure 3:**
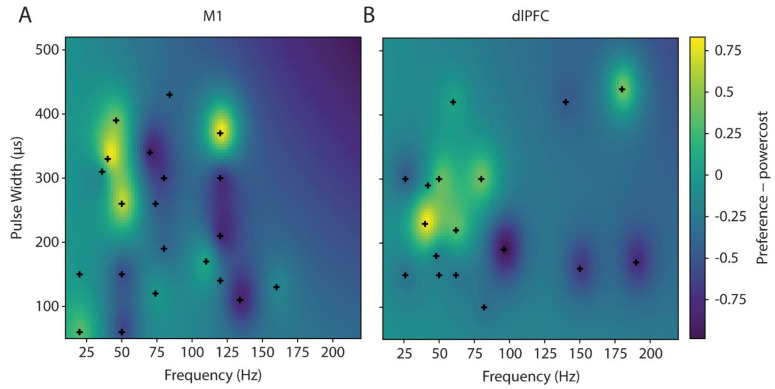
Frequency, pulse width, and amplitude (not shown) optimizations for single electrode combination in M1 on day 3 (**A**) and in dlPFC on day 4 (**B**). Yellower colors indicate preferred stimulation parameters, while bluer colors indicate non-preferred stimulation parameters.

On the third and fourth days, optimal single-site stimulation parameters (frequency, pulse width, and amplitude) were identified separately for the prior contact choices based on the result of a Bayesian parameter space optimization utilizing a probit Gaussian process to assess her preferences, similar to what we have previously described in Zhao et al. [7]. Four initial settings were chosen, from the combination of two frequencies (50 and 20 Hz for M1 and 26 and 50 Hz for dlPFC) and two pulse widths (150 and 60 μs for M1 and 150 and 300 μs for dlPFC). Parameter preference was determined through a two-step process where, for each combination of frequency and pulse width, amplitude was varied, and the patient was asked to choose her preferred amplitude before comparison to previous settings, considering both pain relief and side effects. Parameter rankings were then converted to a list of all pairwise comparisons, where the win and loss was determined by the relative ranking. A probit function was then used to convert the pairwise preferences into relative values for every setting. The values for untested settings were then estimated using Gaussian Process Regression. Subsequent settings to test were selected by generating a random surface from the Gaussian Process Regression model over the range of 50–500 μs and 20–180 Hz, with a penalizing factor for high energy parameters (powercost), and then selecting the setting with the maximum value. A total of 21 settings with 32 comparisons were made for M1 and 17 settings with 72 comparisons for dlPFC (Figure 3). She rated her pain as a 2/10 for the optimal M1 setting and as a 0/10 for the optimal dlPFC setting, with no side effects observed. In both cases, the optimization resulted in good coverage of the parameter settings, but the study was limited by the duration of the clinical visit, and it cannot be shown that the model converged to a global minimum without further testing.

**Figure 4:**
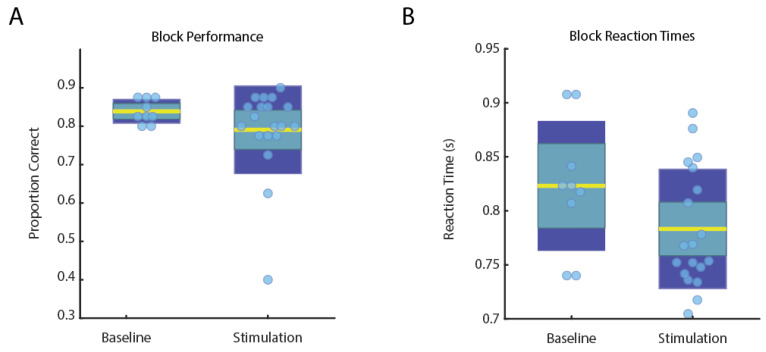
Performance (**A**) and reaction time (**B**) on the 2-back was comparable for baseline (*n* = 9) and dlPFC stimulation (*n* = 19) blocks, suggesting stimulation did not significantly impair cognitive function. Data for each block are indicated by the light-blue circles, standard deviation in dark-blue, standard error in teal, and the mean in yellow.

Due to concerns that dlPFC stimulation might impair cognitive function, working memory was assessed at baseline and with four preferred and two non-preferred dlPFC stimulation settings using the N-back task [15]. The 2-back was chosen as her baseline performance was approximately 80% accurate. In total, she performed 9 blocks at baseline without stimulation and 19 blocks with stimulation. There appeared to be some variability in N-back performance metrics across specific dlPFC stimulation settings (Figure 4). However, a Wilcoxon rank sum test showed that, across all dlPFC stimulation parameters, stimulation did not significantly affect accuracy (*p* = 0.23) or reaction times (*p* = 0.17). While her performance did not change with stimulation, she sometimes reported increased difficulty during stimulation blocks, suggesting that dlPFC stimulation may modulate perceived cognitive effort.

On the fifth day, dual site M1 and dlPFC stimulation was tested in a blinded fashion against previous independent settings. Following extended testing and comparison between relieving parameter combinations, we found that the dual site stimulation reliably provided the most relief compared to either single site in isolation.

### 2.4. Effects of Stimulation on Electrophysiology

**Figure 5:**
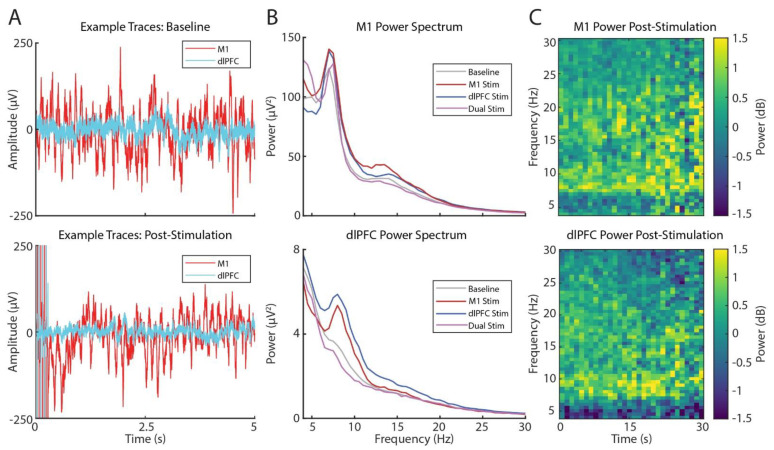
Effects of stimulation on M1 and dLPFC activity. (**A**) Voltage-time traces of M1 (red) and dlPFC activity (cyan) at baseline (top) and post-stimulation (bottom). (**B**) Power spectra at baseline and following M1 (maroon), dlPFC (dark blue), or dual-site stimulation (purple) in M1 (top) and dlPFC (bottom). (**C**) Spectrograms of M1 (top) and dlPFC (bottom) power post-stimulation normalized to the baseline period.

While no seizures were observed at any time during or after stimulation, we were concerned that the stimulation may trigger subclinical epileptiform electrophysiological activity. We reviewed post-stimulation cortical activity and found no significant after-discharges or noticeable difference in activity upon visual review (Figure 5A). Simple threshold-based analysis of broadband signals also did not identify the presence of seizure-like activity. 

While no concerning changes in activity were observed, we noticed some changes in spectral power immediately following stimulation. We combined all post-stimulation recording periods to quantify stimulation-induced changes in cortical activity. Using FieldTrip to calculate power spectra [16], we built several Generalized Linear Mixed-Effects Models (GLMEs, bandPower ~1 + timeWindow + stimulationLocation + (1|channelNumber)) to determine if bandPower in specific brain areas varied with stimulation location, and we found significant (GLME, *p* < 0.05) power changes in theta (6–9 Hz) and low beta (13–20 Hz) bands following stimulation (Table 1 and Figure 5B). Interestingly, individually, M1 and dlPFC stimulation tended to increase theta- and beta-band power at both sites, while dual site stimulation tended to decrease power (GLME, *p* < 0.05). There was weak evidence that theta and beta power changed over time post-stimulation (Figure 5C, GLME, *p* < 0.05), but effect sizes were all below 0.014 μV^2^s^−1^; moreover, both positive and negative effects were observed suggesting these effects are unreliable across recording locations and stimulation parameters. Additionally, we did not find any power effects based on specific stimulation parameters; this may be due to our focus on finding optimized stimulation parameters for pain relief rather than for repeated measures analysis. 

### 2.5. Final Treatment Plan

Overall, pain ratings decreased from a baseline of 8.5/10 to 0/10 following stimulation parameter optimization. Based on this change, she underwent permanent implantation of the cortical stimulation system. Under general anesthesia, the previous post-auricular incision was opened, and the electrode extension leads were disconnected, cut, and carefully removed to avoid contamination. New extensions were connected to both paddles and tunneled subcutaneously to an implantable pulse generator (IPG) placed over the right chest. The settings that provided maximal pain relief were programmed into the IPG, and the system was configured for remote programming to continue the forced-choice Bayesian optimization remotely using NeuroSphere™ (Abbott, Abbott Park, IL, USA). At one-month post-procedure, she reported pain levels no higher than 2/10 at any time, and we began remote programming to continue to search for optimal settings over time using our Bayesian optimization.

## 3. Discussion

CPSP affects between 1 and 12% of stroke patients with higher rates for experiencing pain following strokes in the thalamus and lateral medulla. Despite the prevalence of CPSP, it remains poorly understood and difficult to treat [2].

Off-label neurostimulation therapies, such as motor cortex stimulation, are considered in treatment-resistant cases of CPSP. While the mechanisms underlying the effect of motor cortex stimulation are currently unknown, prior case series have implicated changes in cerebral blood flow and thalamic bursting [2]. Reviews of these case series estimated the 1-year success of motor cortex stimulation to be between 45–50% (2). Despite this, we have not observed efforts to implement a systematic approach to personalize stimulation. Therefore, we implemented a forced-choice Bayesian preference optimization to be used long-term with remote programming during an inpatient externalization where feasibility was demonstrated and encouraging results found.

Noninvasive studies have shown that dlPFC function is abnormally increased in patients with chronic pain, and non-invasive stimulation of the dlPFC has been reported to be effective for alleviating such pain. Converging evidence suggests that dlPFC has a role in cognitive components of pain, acting as an interface between cognitive processing and pain regulation [8]. Considering the inconsistency of motor cortex stimulation and preliminary evidence of dlPFC’s role in pain processing, we elected to target both regions with the aim of treating the patient’s pain in an individualized multifaceted manner. While dlPFC plays a critical role in working memory performance, we did not observe a significant performance decrease in working memory function during various dlPFC stimulation regimes, though there was some variation in task performance with different stimulation parameters. Furthermore, some settings were perceived oddly (e.g., feeling “spacey”), yet were sometimes associated with increases in target or non-target performance. These results imply that dlPFC stimulation may be biasing attention towards or away from certain environmental features (e.g., targets), which may be similarly beneficial for biasing attention away from pain. While the exact mechanism by which the stimulation relieves pain is unknown, the electrophysiology suggests that stimulation produces short-term plastic changes in the recorded networks observed here as changes in theta- (6–9 Hz) and low beta-band (13–20 Hz) power following stimulation. These frequency bands are known to play an important role in interareal communication, attention, and working memory [17,18,19]. Together with electrophysiology demonstrating no significant after discharges or seizure-like activity, these results suggest invasive stimulation of dlPFC may prove to be a promising and safe target for various aspects of chronic, intractable pain.

Our results at first appear inconsistent with the one randomized controlled trial to examine repetitive TMS to the dlPFC for CPSP, which found limited efficacy [20]. However, intracranial stimulation has a more direct effect on the cortical substrate and has been demonstrated to have efficacy in other clinical settings where repetitive TMS has failed [11,12]. Furthermore, this previous study did not pursue an optimization strategy, which we view as central to our outcome.

We observed that dual dlPFC and M1 stimulation efficacy was greater than M1 or dlPFC stimulation alone, suggesting a promising new approach for treating CPSP. Dual stimulation was also associated with a qualitatively different spectral power response: a decrease in theta and beta power in both regions, in contrast to the increase seen with single site stimulation. The current case demonstrates the significant heterogeneity in response dependent on the settings chosen, motivating the necessity of methods, such as Bayesian parameter space optimization, to ensure treatment is accurately tailored to a patient’s condition. Additionally, newer stimulation systems have features for remote programming, providing a platform by which parameters could be continuously optimized away from the clinic via a simple video visit [21]. The ability to identify multiple, similarly effective yet largely different parameter combinations will provide patients and clinicians with more flexibility as preferences and symptoms vary, which we hypothesize may improve long-term outcomes. Exploring new targets and stimulation settings prompts us to also consider the potential side effects, such as impairments in cognition or seizure generation, for which quantitative behavioral testing and electrophysiology can provide helpful platforms.

## 4. Conclusions

We provide a single case report demonstrating the possible utility of Bayesian preference parameter space optimization and a dual site, M1 and dlPFC, stimulation protocol to treat CPSP through a single burr hole. The approach of dual-site stimulation in dlPFC and M1 may be considered for treatment of patients with CPSP who do not respond to conventional pharmaceuticals and noninvasive procedures. The combination of both sites and systematic parameter optimization are associated with significant reductions in pain without evidence of side effects or reductions in cognitive performance.

## Figures and Tables

**Table 1:** GLME results showing effect of stimulation on theta- (6–9 Hz) and low beta-band (13–20 Hz) power.

Stim Target	Power Band	Recording Location	*p*-Value	Effect Size (μV^2^)
M1	Theta	M1	0	23.8
M1	Theta	dlPFC	0	2.22
M1	Beta	M1	1.21 × 10^−12^	3.30
M1	Beta	dlPFC	0.115	−0.0667
dlPFC	Theta	M1	0	13.4
dlPFC	Theta	dlPFC	0	3.87
dlPFC	Beta	M1	9.97 × 10^−2^	0.613
dlPFC	Beta	dlPFC	0	0.471
M1/dlPFC	Theta	M1	5.84 × 10^−3^	2.69
M1/dlPFC	Theta	dlPFC	0	−1.17
M1/dlPFC	Beta	M1	0	−4.89
M1/dlPFC	Beta	dlPFC	7.59 × 10^−8^	−0.170

## Data Availability

The data presented in this study are available on request from the corresponding author.
